# Detection of infectious organisms in archival prostate cancer tissues

**DOI:** 10.1186/1471-2407-14-579

**Published:** 2014-08-09

**Authors:** Melissa A Yow, Sepehr N Tabrizi, Gianluca Severi, Damien M Bolton, John Pedersen, Anthony Longano, Suzanne M Garland, Melissa C Southey, Graham G Giles

**Affiliations:** Genetic Epidemiology Laboratory, Department of Pathology, University of Melbourne, Melbourne, VIC 3010 Australia; Molecular Microbiology Laboratory, Department of Microbiology and Infectious Diseases, Bio 21 Institute, Royal Women’s Hospital, Parkville, VIC 3052 Australia; Department of Obstetrics and Gynaecology, University of Melbourne, Melbourne, VIC 3010 Australia; Cancer Epidemiology Centre, Cancer Council Victoria, Melbourne, VIC 3004 Australia; Centre for Epidemiology and Biostatistics, School of Population Health, University of Melbourne, Melbourne, VIC 3010 Australia; Department of Surgery, University of Melbourne, Austin Health, Heidelberg, VIC 3084 Australia; TissuPath, Mount Waverley, VIC 3049 Australia; Department of Anatomical Pathology, Monash Medical Centre, Clayton, VIC 3168 Australia

**Keywords:** Prostate cancer, Sexually transmitted infection, Infection, qPCR

## Abstract

**Background:**

Seroepidemiological studies have reported associations between exposure to sexually transmitted organisms and prostate cancer risk. This study sought DNA evidence of candidate organisms in archival prostate cancer tissues with the aim of assessing if a subset of these cancers show any association with common genital infections.

**Methods:**

221 archival paraffin-embedded tissue blocks representing 128 histopathologically confirmed prostate cancers comprising 52 “aggressive” (Gleason score ≥ 7) and 76 “non-aggressive” (Gleason score ≤ 6) TURP or radical prostatectomy specimens were examined, as well as unaffected adjacent tissue when available. Representative tissue sections were subjected to DNA extraction, quality tested and screened by PCR for HSV-1, HSV-2, XMRV, BKV, HPV, *Chlamydia trachomatis, Ureaplasma parvum, Ureaplasma urealyticum, Mycoplasma genitalium,* and *Trichomonas vaginalis*.

**Results:**

195 of 221 DNA samples representing 49 “aggressive” and 66 “non-aggressive” prostate cancer cases were suitable for analysis after DNA quality assessment. Overall, 12.2% (6/49) aggressive and 7.6% (5/66) non-aggressive cases were positive for any of the candidate organisms. *Mycoplasma genitalium* DNA was detected in 4/66 non-aggressive, 5/49 aggressive cancers and in one cancer-unaffected adjacent tissue block of an aggressive case. *Ureaplasma urealyticum* DNA was detected in 0/66 non-aggressive and 1/49 aggressive cancers and HSV DNA in 1/66 non-aggressive and 0/49 aggressive cancers. This study did not detect BKV, XMRV, *T. vaginalis*, *U. parvum*, *C. trachomatis* or HPV DNA.

**Conclusions:**

The low prevalence of detectable microbial DNA makes it unlikely that persistent infection by the selected candidate microorganisms contribute to prostate cancer risk, regardless of tumour phenotype.

## Background

The infection hypothesis for prostate cancer was first proposed in the mid-twentieth century [1]. Subsequently, many studies have sought associations between sexually transmitted infections (STIs) and prostate cancer risk but no clear association with a pathogen has been established. A meta-analysis of 29 case–control studies (1966–2003) reported associations between prostate cancer risk and any STI (OR 1.48 95% CI 1.26-1.73), gonorrhoea (OR 1.35 95% CI 1.05-1.83), and HPV (OR 1.39 95% CI 1.12-2.06) [2]. Recently, large prospective sero-epidemiological studies examining the associations between seropositivity to infectious agents and prostate cancer [3, 4] have reported only modest associations between positive serology and prostate cancer.

There is also growing evidence of associations between prostate cancer risk and variants in genes involved in the response to infection and inflammation. Common genetic variants of genes functionally linked to inflammation and immunity such as COX-2 [5], RNASEL [6] and TLR4 [7] have been associated with prostate cancer risk suggesting that infection and host response to infection may be involved in its development.

Case–control studies nested within large prospective seroepidemiological cohort studies have reported only modest associations between evidence of exposure to common STIs and prostate cancer risk (*T. vaginalis* OR 1.43 95% CI 1.00-2.03) [3] or no association (HPV-33 OR 1.14, 95% CI 0.76-1.72; *C. trachomatis* OR 1.13, 95% CI 0.65-1.96) [4]. It is likely that these studies would have been limited by the biases inherent in the measures of exposure applied. Serological methods to measure past infection by organisms such as *C. trachomatis*, *N. gonorrhoea* and HPV may underestimate actual exposure due to poor sensitivity. Kirnbauer et al. [8] demonstrated that only 59% of those positive for HPV16 DNA at the cervix produced a measureable serological response. The low sensitivity of serological assays may be due to the waning of antibody titres over time. In addition, the time to seroconversion may be lengthy and those infected may not seroconvert at all [9].

It has also been suggested that these studies may have been prone to misclassification bias, due to the widespread use of prostate specific antigen (PSA) testing as a screening device for prostate cancer within the study period. This may have led to the inclusion of subclinical slow-growing prostatic neoplasms that diminished their ability to detect meaningful associations between measures of exposure and clinically significant phenotypes. Therefore, in the current environment with respect to PSA screening, studies should incorporate subgroup analysis into their design in order to discriminate factors that may influence the aetiology or progression of clinically relevant tumours from indolent phenotypes [10].

We examined archival tissue from aggressive and non-aggressive prostate cancer phenotypes and used semi-quantitative molecular methods to seek evidence of infection by common sexually transmitted or other organisms at the tissue level.

We hypothesised that the prevalence of DNA from *C. trachomatis*, *U. urealyticum*, *U. parvum*, *T. vaginalis*, *M. genitalium*, herpes simplex virus (HSV) 1 and 2, BK virus, Xenotropic murine leukemia virus-related virus (XMRV), and human papillomavirus (HPV), was the same across tumour phenotypes (non-aggressive and aggressive prostate cancer). We screened samples against a panel of sexually transmitted and other infectious organisms to determine prevalence according to tumour phenotype.

## Methods

Cases were drawn from three existing prostate cancer research projects, (1) the Melbourne Collaborative Cohort Study (MCCS) [11], a population-based prospective cohort study, recruited over the period 1990–1994, (2) the Risk Factors for Prostate Cancer Study (RFPCS) [12], a population-based case control study and (3) the Early Onset Prostate Cancer Study (EOPCS) [13], a population based case series of males diagnosed with prostate cancer aged ≤56 years of age. Approval for use of the samples arising from these studies was given by the Human Research Ethics Committee of Cancer Council Victoria.

Specimens were selected on the basis of Gleason score [14] determined by review of diagnostic haemotoxylin and eosin stained slides by a single pathologist (JP). Aggressive and non-aggressive tumours were compared. Aggressive tumours were defined as Gleason score ≥7, poorly-differentiated, including tumours staged at T4, N + (lymph node positive), or M + (distant metastases) regardless of their Gleason score or grade of differentiation. Non-aggressive tumours were defined as well-differentiated with a Gleason score ≤6.

We used archival prostate tissues resected from men that had undergone either radical prostatectomy (RP) or transurethral resection of the prostate (TURP) within the period 1992–2005. A total of 221 formalin-fixed paraffin-embedded tissue blocks (including unaffected adjacent tissue when available) representing 128 histopathologically confirmed prostate cancers comprising TURP and RP specimens were examined.

We processed formalin-fixed, paraffin-embedded radical prostatectomy and TURP specimens using the sandwich sectioning method [15]. To minimize cross-contamination between the samples, gloves and the microtome blade were changed and the microtome washed with histolene, bleach, and 80% ethanol between each sample. Formalin-fixed paraffin-embedded breast tissue was sectioned between every four prostate tissue blocks to ensure no carry-over of DNA. The outer three-micrometer sections were stained with haematoxylin and eosin and validated by a single pathologist to confirm the presence of cancer and the initial histological diagnosis (AL). The four inner seven-micrometer sections remained unstained and were utilised for DNA extraction and molecular assays.

Sections selected for DNA extraction were deparaffinised with histolene and absolute ethanol and the tissue pellet air-dried. Digestion of the tissue was achieved by resuspending the pellet in 160 μL Tissue Lysis Buffer (Roche, Australia) and 40 μL proteinase K (Roche, Australia) and incubating overnight in a heat block at 37°C. A 200 μL volume of lysate was extracted using the MagNA Pure LC instrument and MagNA Pure LC DNA Isolation Kit I (Roche, Australia) with an elution volume of 100 μL as per the manufacturer’s protocol.

Integrity of the DNA extracted from prostate tissue was ascertained by amplification of a 268 bp region of the human beta-globin gene as previously described [16].

We qualitatively screened samples for *Chlamydia trachomatis* by the COBAS® TaqMan® CT Test, v2.0 (Roche, Australia). Amplification and detection of HPV on all samples was carried out using the PapType High-Risk (HR) HPV Detection and Genotyping kit (Genera Biosystems, Melbourne, Victoria, Australia) [17]. In addition, 49 aggressive cases were screened by DNA ELISA kit HPV SPF10, version 1 (Labo Bio-medical Products BV, Rijswijk, The Netherlands) according to the manufacturer’s instructions. Published primers, probes and Real-Time PCR protocols for *Ureaplasma urealyticum* [18], *Ureaplasma parvum* [18], *Mycoplasma genitalium* [19], *Trichomonas vaginalis* [20, 21], Xenotropic Murine Retrovirus [22], BK virus [23] AND HSV [24] were applied to the screening of samples with minor modifications (Table 1). Assays to detect *T. vaginalis* and HSV 1 and 2 were performed on the LightCycler Carousel (Roche, Australia) and all other assays on the LightCycler 480 (Roche, Australia).

**Table 1:** **Primers, probes and commercial kits used in this study for detection, quantification and genotyping**

Organism	Target	Primers and probes (5′ to 3′)		Product size	References
*C. trachomatis*	CT cryptic plasmid			206 bp	COBAS ® TaqMan ® CT test, v2.0, Roche
*U. urealyticum*	*ureB* gene	UUureF	GATCACATTTCCACTTATTTGAAACA	100 bp	Mallard et al. [18]
		UUureR	AAACGACGTCCATAAGCAACTTTA		
		UUure2MGB	AAACGAAGACAAAGAAC		
*U. parvum*	*ureB* gene	UPureF	GATCACATTTTCACTTGTTTG AAGTG	99 bp	Mallard et al. [18]
		UPureR	AACGTCGTCCATAAGCAACTTTG		
		UPure1MGB	AGGAAATGAAGATAAAGAAC		
*M. gentalium*	MgPa adhesin gene	MgPa-355 F	GAGAAATACCTTGATGGTCAGCAA	78 bp	Jensen et al. [19]
		MgPa-432R	GTTAATATCATATAAAGCTCTACCGTTGTTATC		
		MgPa-380	FAM-ACTTTGCAATCAGAAGGT-MGB		
HPV				140-150 bp	Genera Biosystems Ltd
HSV-2	Glycoprotein D gene	H5	TGTGCTATCCCCATCACGGT	239 bp	Powell et al. [24]
		H6	GGCTCGGTGCTCCAGGATAA		
		HSVgs-1	CCGCTGGAACTACTATGACAGCTTCAGC		
		HSVgs-2	CCGTCAGCGAGGATAACCTGGG		
XMRV	Integrase gene	XMRV4552F	CGAGAGGCAGCCATGAAGG	122 bp	Schlaberg et al. [22]
		XMRV4653R	GAGATCTGTTTCGGTGTAATGGAAA		
		XMRV4673R	CCCAGTTCCCGTAGTCTTTTGAG		
		XMRV4572MGB	6FAMAGTTCTAGAAACCTCTACACTCMGBNFQ		
BK virus	TAg	BK-Hirsch-1	AGCAGGCAAGGGTTCTATTACTAAAT	128 bp	Hirsch et al. [23]
		BK-Hirsch-2	GAAGCAACAGCAGATTCTCAACA		
		Probe	HEXAAGACCCTAAAGACTTTCCCTCTGATCTACACCAGTTTBHQ1		
*T. vaginalis*	A6p region	TVA5	GATCATGTTCTATCTTTTCA	102 bp	Riley et al. [20], Tabrizi et al. [21]
		TVA6	GATCACCACCTTAGTTTACA		
		TV-F1AS	TTACACTCTGAGTTCTTTCTTCTA		
		TV-F2AS	AGTCTTTTTTAGATTTTGAAACA		
Human β-globin	β-globin gene	GH20	GAAGAGCCAAGGACAGGTAC	268 bp	Resnick et al. [16]
		PC04	CAACTTCATCCACGTTCACC		

## Results and discussion

Of the 221 samples, 195 (88.2%) produced a 268 bp product of the human beta-globin gene in quality control PCR testing and were deemed suitable for further analysis. Of these, 49 cases were classified as aggressive and 66 cases as non-aggressive. Of the 49 aggressive cases, 13 cases also had an adjacent normal tissue block. Of the 66 non-aggressive cases, 38 had both a tumour and normal block available.

Table 2 shows the prevalence of *M. genitalium, U. urealyticum*, and HSV (7.8%, <1% and <1% respectively) and that no difference in prevalence between aggressive and non-aggressive phenotypes was observed. Herpes simplex virus (indeterminate type) DNA was detected in 1/66 non-aggressive prostate cancer tissues and in none of 49 aggressive prostate cancer tissues. *Mycoplasma genitalium* DNA was detected in 4/66 (6.0%) non-aggressive, 5/49 (10.2%) aggressive and in one cancer-unaffected tissue block of an aggressive case. *Ureasplasma urealyticum* DNA was detected in none of the non-aggressive and 1/49 (2.0%) aggressive prostate cancer cases. *Ureaplasma parvum*, *T. vaginalis*, *C. trachomatis*, BKV, XMRV or HPV DNA was not detected in any prostate cancer tissue screened in this study.

**Table 2:** **Identification of infectious organisms in archival prostate cancer tissue**

			Overall prevalence			
Organism	Aggressive cases	Non-aggressive cases	Tumour tissue		“Normal” tissue ^b^	
	n = 49	n = 66	n = 115	%	n = 51	%
HSV	0	1	1	0.87	0	0
*Mycoplasma genitalium*	5	4	9	7.83	1	1.96
*Ureaplasma urealyticum*	1	0	1	0.87	0	0
Other^a^	0	0	0	0	0	0

P-values from Fisher exact test comparing the prevalence of each infectious organism between aggressive and non-aggressive samples and between tumour and normal tissue samples are all greater than 0.18.

^a^Other includes *U. parvum*, *T. vaginalis*, *C. trachomatis*, BKV, HPV and XMRV.

^b^Adjacent tissue with no histological evidence of cancer.

Our negative findings with respect to the presence of viral DNA in formalin-fixed prostate cancer tissues are consistent with those of Bergh et al. [25] who screened 352 formalin-fixed paraffin embedded tissues of benign prostatic hyperplasia cases for evidence of HSV 1 and 2, BKV or HPV infection and detected no viral DNA. In addition, Martinez-Fierro and colleagues [26] reported a low and insignificant prevalence of XMRV and BKV DNA in fresh frozen prostate material but reported a positive association between prostate cancer and HPV prevalence (OR 3.98, 95% CI 1.17-13.56, p = 0.027), in contrast to our study that did not detect HPV DNA in any prostate sample.

One of the weaknesses of our study is the limited statistical power to detect moderate differences in the prevalence of infectious organisms due to the low prevalence we observed in all our samples. For example, for M. genitalia, the most prevalent organism in our samples, the statistical power to detect a four-fold higher prevalence in tumour tissue samples than in normal tissue samples (i.e. 8% vs 2%) at a 0.05 level of statistical significance was lower than 50%.

## Conclusions

The methods we employed for this study were direct and robust with respect to sensitivity and specificity for the target organisms. We chose primers that generated small amplimers (≤268 bp) to account for fragmentation of the DNA extracted from formalin-fixed paraffin embedded tissues. We conclude that it is unlikely that the microorganisms which were included in the candidate panel contributed to the development of prostate cancer in our Australian sample of prostate cancers due to the low prevalence or complete absence of detectable microbial DNA in the tissue samples. Our study hypothesis and aims assumed persistent infection with the candidate organisms allowing for molecular detection in the FFPE material. We cannot exclude the possibility of an initial infection leading to oncogenic sequelae followed by clearance either by natural immunity or administration of antibiotics.

## Abbreviations

BKV: BK virus
DNA: Deoxyribonucleic acid
EOPCS: Early onset prostate cancer study
FFPE: Formalin-fixed paraffin-embedded
HPV: Human papillomavirus
HSV-1: Herpes simplex virus 1
HSV-2: Herpes simplex virus 2
MCCS: Melbourne Collaborative Cohort Study
PCR: Polymerase chain reaction
PSA: Prostate specific antigen
qPCR: Quantitative polymerase chain reaction
RP: Radical prostatectomy
RFPCS: Risk factors for prostate cancer study
STI: Sexually transmitted infection
TURP: Transurethral resection of the prostate
XMRV: Xenotropic murine leukemia virus-related virus.
